# Causes of death following small cell lung cancer diagnosis: a population-based analysis

**DOI:** 10.1186/s12890-022-02053-4

**Published:** 2022-07-04

**Authors:** Xue-qin Wu, Jing-yi Li, Wen-jing Du

**Affiliations:** 1 Department of Radiotherapy, Shanxi Province Cancer Hospital, Taiyuan, 030000 Shanxi China; 2 Department of Radiotherapy, Shanxi Hospital Affiliated to Cancer Hospital, Chinese Academy of Medical Sciences, Taiyuan, 030000, Shanxi China; 3 Department of Radiotherapy, Cancer Hospital Affiliated to Shanxi Medical University, Taiyuan, 030000 Shanxi China

**Keywords:** Cause of death, SCLC, SEER, Lung cancer

## Abstract

**Purpose:**

To examine the distribution of causes of death (CODs) in patients with small cell lung cancer (SCLC).

**Methods:**

Patients diagnosed with SCLC were identified from the Surveillance, Epidemiology, and End Results Program database during 2004–2015. Standardized mortality rates (SMRs) were performed for each COD to present changes in risk for a particular COD following SCLC diagnosis.

**Results:**

A total of 44,506 patients diagnosed with SCLC were identified in this study, and 42,476 patients died during the follow-up. Of total deaths, 69.5% occurred within the first years after diagnosis, 26% occurred from 1 to 3 years, and 4.5% individuals survived longer than 3 years. In addition, 88.7% of deaths were caused by SCLC, followed by non-cancer causes (7.1%) and other cancers (4.2%). Moreover, non-cancer CODs increased from 6.3 to 30% over time after 3 years of diagnosis. As for non-cancer CODs, cardiovascular diseases, COPD, and septicemia were the most common in SCLC.

**Conclusion:**

Non-cancer CODs, such as cardiovascular events, COPD and septicemia, contribute to a considerable proportion of deaths among long-term SCLC survivors, supporting the involvement of multidisciplinary care for the follow-up strategy in SCLC.

**Supplementary Information:**

The online version contains supplementary material available at 10.1186/s12890-022-02053-4.

## Introduction

Lung cancer is the leading cause of cancer-related deaths worldwide. Small cell lung cancer (SCLC) as a high-grade neuroendocrine tumor, accounts for approximately 15% of all diagnosed lung cancers [1]. Characterized by rapid growth and early tendency to widespread metastasis, 70% of SCLC patients are initially diagnosed at an advanced stage [2]. In addition, different from NSCLC, surgery is not appropriate for most SCLC, while chemo- and radiotherapy represent the mainstay of treatment. Though a good initial response to such treatment is observed, most of the patients might experience relapse with the disease being refractory, leading to a very dismal prognosis [3, 4]. In recent years, with the emergence of multidisciplinary treatment strategies, including concurrent chemoradiotherapy, prophylactic cranial irradiation, targeted and immunotherapy, the prognosis of SCLC has improved with a 2-year overall survival (OS) of 20–54.4% and 2.8–19.5% for limited-stage and extensive-stage disease, respectively [5–8].

Recently, increasing studies reported that owing to the improved survival among cancer patients, patients are more likely to live long enough after initial diagnosis to the point that non-cancer-related comorbidities may considerably affect their overall survival [9–12]. However, due to the low survival rate, no study has formally evaluated the characteristics of long-term (3-year) survivors of SCLC [13]. For SCLC, tobacco use is found to be closely related to its tumorigenesis, with about 95% of patients being current or former smokers. Patients with SCLC are more likely to suffer from smoking-related chronic comorbidities, such as chronic obstructive pulmonary disease (COPD), cardiovascular and cerebrovascular diseases [14]. In addition, most patients diagnosed with SCLC are over 60 years old, where studies found that the increase in age is also accompanied by an increase in comorbidity [15, 16]. Therefore, understanding the distribution of different cause of death (COD) in patients with SCLC is important for developing individual follow-up strategies.

In this study, an analysis of CODs after a diagnosis of SCLC was conducted, aiming to fill the knowledge gap in the prevention of potential underlying diseases that may lead to death.

## Material and methods

### Study population

Patients with SCLC diagnosed between 2004 and 2015 were identified from the Surveillance, Epidemiology, and End Results (SEER) registries. SCLC was defined based on the following International Classification of Diseases for Oncology Third Edition (ICD-O-3), morphology codes: 8002/3, 8041/3, 8042/3, 8043/3, 8044/3, and 8045/3. Patients with more than one cancer and those younger than 18 years were excluded. SEER does not uncover sensitive patient information, and we registered the study with the Institutional Review Board (IRB) and received clearance.

### Statistical analysis

CODs were categorized by the International Classification of Diseases (ICD)‑10 codes and were listed in Additional file 1: Table S1. For included patients with SCLC, we surveyed CODs with further stratification by these variables: age at diagnosis, race, treatment (chemotherapy and radiotherapy) and stage. Data are presented in 3 groups based on the latency period: < 1 year, 1–3 years, and > 3 years following SCLC diagnosis.

Standardized mortality ratios (SMRs) with 95% confidence intervals (CIs) were computed for each specific COD using SEER*Stat 8.3.9. SMR was defined as the observed number of deaths in the included patients divided by the expected number of deaths in the matched general population (adjusting for sex, age, race and calendar year). The SMR in this study provides the excess mortality of a specific COD after a diagnosis of SCLC relative to the background mortality in the US. A significant increase in the risk of a specific COD was defined as a *p* value < 0.05. All statistical tests were 2-sided.

## Results

### Baseline characteristics

A total of 44,506 patients diagnosed with SCLC were identified from 2004 to 2015, of which, 74.4% were aged over 60 years old, 86.2% were white individuals, and 64.5% were initially diagnosed as stage IV. Surgery was performed in less than 3% of patents. During the follow-up, 42,476 patients died with a median age of 66.75 years old. Of total deaths, 69.5% occurred within the first years after diagnosis, 26.0% occurred from 1 to 3 years, and 4.5% individuals survived longer than 3 years (Table 1). In addition, 88.7% of death were caused by SCLC, followed by non-cancer causes (7.1%) and other cancers (4.2%). About 34 077 patients received at least one treatment type for SCLC (surgery, radiotherapy, chemotherapy). Among them, 28 847 (84.7%) died from SCLC, and 137 (0.4%) died from other smoking related cancers (cancers of the esophagus, larynx, mouth, throat, kidney, bladder, liver, pancreas, stomach, cervix, colon, and rectum, as well as acute myeloid leukemia). Of patients who were not treated, 84.7% also died from SCLC (8841), and nearly 1% died from other smoking-related cancers (100). Moreover, among patients who died from other cancers, receiving treatment led to less likely to die from smoking-related cancers than no treatment (12.0% vs. 15.6%).

**Table 1:** Baseline characteristics of patients with SCLC

Characteristic	Diagnosed cases, n	Deaths, n	Mean age at death, y	Deaths by time after diagnosis, n (%)		
				< 1 y	1–3 y	> 3 y
All patients	44,506	42,476	66.75	29,536 (69.5)	11,040 (26.0)	1900 (4.5)
*Sex*						
Male	22,521	21,606	66.36	15,662 (72.5)	5173 (23.9)	771 (3.6)
Female	21,985	20,870	67.14	13,874 (66.5)	5867 (28.1)	1129 (5.4)
*Age at diagnosis, y*						
< 60	11,389	10,566	53.41	6434 (60.9)	3558 (33.7)	574 (5.4)
60–69	15,496	14,731	64.66	9873 (67.0)	4129 (28.0)	729 (4.9)
≥ 70	17,621	17,179	76.74	13,229 (77.0)	3353 (19.5)	597 (3.5)
*Race*						
White	38,373	36,684	66.85	25,603 (69.8)	9458 (25.8)	1623 (4.4)
Black	4512	4306	65.01	2920 (67.8)	1180 (27.4)	206 (4.8)
Other	1621	1486	69.37	1013 (68.2)	402 (27.1)	71 (4.8)
*Cancer stage*						
I–III	15,591	14,180	6721	7574 (53.4)	5176 (36.5)	1430 (10.1)
IV	28,915	28,296	6652	21,962 (77.6)	5864 (20.7)	470 (1.7)
*Treatment*						
Surgery	1114	877	6620	339 (38.7)	386 (44.0)	152 (17.3)
Radiotherapy	20,498	19,028	6454	10,586 (55.6)	6957 (36.6)	1485 (7.8)
Chemotherapy	31,154	29,408	6507	17,711 (60.2)	9979 (33.9)	1718 (5.8)

**Fig. 1:**
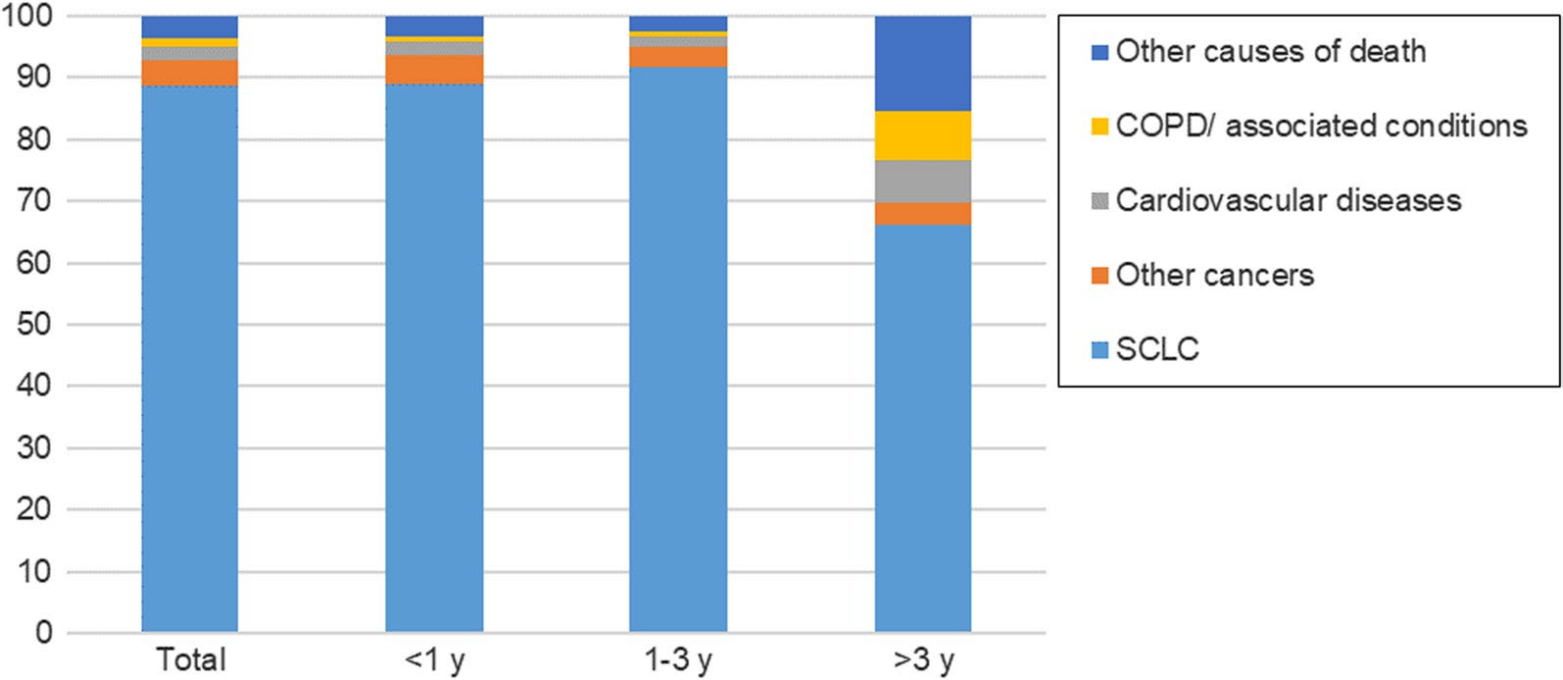
Causes of death (CODs) after small cell lung cancer (SCLC) within each latency period

As for non-cancer CODs, cardiovascular disease was the most common one (30.5%), followed by COPD (17.5%) (Fig. 1). Although patients with SCLC were at a statistically significant increased risk of death from each specific type of non-cancer cause (except for Alzheimer’s disease), the SMR was greatest for septicemia and COPD deaths at 9.47 (95% CI, 8.02–11.10) and 7.59 (95% CI, 6.96–8.26), respectively (Table 2). In addition, with increasing age at diagnosis, the extent of SMR elevation was attenuated considerably on a relative scale for most CODs, partly corresponding to increased background deaths occurring with aging. Patients with stage I-III were more likely to die from other causes (12.49% vs. 9.77%), while patients with stage IV were more likely to die from SCLC (78.46% vs. 88.09%). Additional file 2: Table S2–Additional file 11: Table S11 showed the analysis across variant demographic and tumor-related subgroups.

**Table 2:** Observed deaths and SMRs for causes of death after diagnosis of SCLC

	Deaths by time after diagnosis							
	< 1 y		1–3 y		> 3 y		Total deaths	
	Observed, no. (%)	SMR (95% CI)	Observed, no. (%)	SMR (95% CI)	Observed, no. (%)	SMR (95% CI)	Observed, no. (%)	SMR (95% CI)
*Cause of death*								
All	29,536 (100)	54.96 (54.33–55.59)*	11,040 (100)	43.00 (42.20–43.81)*	1 900 (100)	8.19 (7.83–8.57)*	42,476 (100)	41.39 (41.00–41.79)*
SCLC	26,306 (89.1)	583.5 (576.5–590.6)*	10,115 (91.6)	475.8 (466.6–485.2)*	1258 (66.2)	73.55 (69.55–77.7)*	37,679 (88.7)	451.5 (447.0–456.1)*
Other cancers	1355 (4.6)	12.56 (11.90–13.25)*	358 (3.2)	6.86 (6.17–7.61)*	67 (3.5)	1.49 (1.16–1.90)*	1780 (4.2)	8.68 (8.29–9.10)*
Noncancer causes	1875 (6.3)	4.88 (4.66–5.10)*	567 (5.1)	3.09 (2.84–3.36)*	575 (30.3)	3.38 (3.11–3.67)*	3017 (7.1)	4.09 (3.94–4.24)*
Septicemia	106 (5.7)	12.76 (10.45–15.44)*	29 (5.1)	7.15 (4.79–10.26)*	17 (3.0)	4.60 (2.68–7.37)*	152 (5.0)	9.47 (8.0211.10)*
Infectious/parasitic diseases including HIV infection	56 (3.0)	10.52 (7.94–13.66)*	4 (0.7)	1.55 (0.42–3.97)	9 (1.6)	4.29 (1.96–8.13)*	69 (2.3)	6.90 (5.37–8.73)*
Diabetes mellitus	34 (1.8)	1.89 (1.31–2.64)*	6 (1.1)	0.69 (0.25–1.51)	9 (1.6)	1.2 (0.55–2.27)	49 (1.6)	1.43 (1.06–1.89)*
Alzheimer’s disease	7 (0.4)	0.5 (0.20–1.03)	4 (0.7)	0.57 (0.16–1.46)	26 (4.5)	3.17 (2.07–4.65)*	37 (1.2)	1.27 (0.89–1.75)
Cardiovascular diseases	593 (31.6)	4.09 (3.77–4.44)*	200 (35.3)	3.0 (2.59–3.44)*	128 (22.3)	2.14 (1.79–2.55)*	921 (30.5)	3.40 (3.18–3.62)*
Cerebrovascular diseases	75 (4.0)	2.65 (2.09–3.32)*	29 (5.1)	2.18 (1.46–3.14)*	45 (7.8)	3.63 (2.65–4.86)*	149 (4.9)	2.76 (2.34–3.24)*
Pneumonia and influenza	76 (4.1)	6.89 (5.43–8.62)*	15 (2.6)	2.94 (1.65–4.85)*	22 (3.8)	4.65 (2.92–7.05)*	113 (3.7)	5.42 (4.47–6.51)*
COPD/ associated conditions	292 (15.6)	8.09 (7.19–9.07)*	86 (15.2)	4.91 (3.93–6.06)*	151 (26.3)	9.38 (7.94–11.00)*	529 (17.5)	7.59 (6.96–8.26)*
Chronic liver disease/cirrhosis	15 (0.8)	2.19 (1.22–3.61)*	4 (0.7)	1.17(0.32–2.99)	6 (1.0)	2.11 (0.77–4.59)	25 (0.8)	1.90 (1.23–2.81)*
Nephritis, nephrotic syndrome, and nephrosis	31 (1.7)	2.89 (1.97–4.11)*	12 (2.1)	2.35 (1.21–4.10)*	11 (1.9)	2.37 (1.18–4.23)*	54 (1.8)	2.64 (1.98–3.44)*
Accidents and adverse effects of medications	70 (3.7)	4.57 (3.56–5.77)*	29 (5.1)	3.85 (2.58–5.53)*	34 (5.9)	5.07 (3.51–7.08)*	133 (4.4)	4.50 (3.77–5.33)*
Suicide and self-inflicted injury	23 (1.2)	5.45 (3.45–8.17)*	8 (1.4)	3.98 (1.72–7.85)*	2 (0.3)	1.27 (0.15–4.57)	33 (1.1)	4.23 (2.91–5.93)*
Other	497 (26.5)	6.10 (5.57–6.66)*	141 (24.9)	3.50 (2.95–4.13)*	115 (20.0)	2.89 (2.39–3.47)*	753 (25.0)	4.66 (4.33–6.01)*

*Indicated *p* < 0.05

### Cause of death within 1 year following SCLC diagnosis

A total of 29,536 death occurred within 1 year after the initial diagnosis of SCLC. 89.1% died of SCLC, 4.6% died of other cancers, and 6.3% died of non-cancer CODs. For non-cancer CODs, the leading causes were cardiovascular diseases (31.6%), COPD (15.6%), and septicemia (5.7%), respectively. In addition, the risks of SCLC patients dying from septicemia (SMR, 12.76; 95% CI, 10.45–15.44), infectious/parasitic diseases (SMR, 10.52; 95% CI, 7.94–13.66), COPD (SMR, 8.09; 95% CI, 7.19–9.07), pneumonia and influenza (SMR, 6.89; 95% CI, 5.43–8.62), and suicide (SMR, 5.45; 95% CI, 3.45–8.17) were 5 times higher than what expected in the general population.

Among patients younger than 60 years of age at diagnosis, the most common non-cancer COD within one year was cardiovascular diseases (35.12%), and the other four accounted for similar proportions (accidents and adverse effects of medications, infectious/parasitic diseases, septicemia, and COPD) (Additional file 2: Table S2). For patients aged more than 60 years, COPD emerged as the second most common non-cancer COD. And the SMR of COPD also became the second-highest one for patients aged more than 70 years (SMR, 6.79; 95% CI, 5.82–7.86) (Additional file 3: Table S3, Additional file 4: Table S4).

### Cause of death within 1–3 years following SCLC diagnosis

A total of 11,040 death occurred within 1–3 years following SCLC diagnosis, 91.6% died of SCLC, 3.2% died of other cancers, and 5.1% died of non-cancer causes. The most common non-cancer CODs continued to be cardiovascular diseases (35.3%). Similar trends were observed across various demographic and tumor-related subgroups, with cardiac diseases being the most common non-cancer COD. (Additional file 2: Tables S2– Additional file 9: Table S9).

### Cause of death within more than 3 years following SCLC diagnosis

After 3 years of survival after SCLC diagnosis, 1 900 patients died. Of them, 66.2% died of SCLC, 3.5% died of other cancers, and 30.3% died of non-cancer causes. The most common non-cancer CODs were COPD (26.3%), cardiovascular diseases (22.3%), and cerebrovascular diseases (7.8%), respectively. The SMR elevated to the highest level for cerebrovascular diseases, COPD and accidents and adverse effects of medication death (Table 2). Similar trends were noticed in patients aged more than 60 years, where COPD was found to be the leading non-cancer COD (Additional file 3: Table S3 and Additional file 4: Table S4).

## Discussion

The cohort study showed that the majority of deaths in US patients with SCLC between 2004 and 2015 occurred within 3 years following diagnosis, most of which were attributed to SCLC. However, due to the significant increase in non-cancer CODs, the frequency of SCLC-related death decreased over time. Among patients who survived more than 3 years, the incidence of non-cancer CODs reached 30.3%. Furthermore, the most common non-cancer CODs in SCLC were cardiovascular diseases, septicemia, COPD, and cerebrovascular diseases, respectively. Of these, cardiovascular diseases and COPD remained dominant even though non-cancer CODs changed over time. In addition, compared with the general U.S. population, patients with SCLC were at a higher risk of dying from most CODs.

Heart disease and cancer are the leading causes of mortality worldwide. Studies assessing the causes of death among cancer patients have revealed an increased risk of cardiovascular diseases [17, 18]. In the previous analysis of lung cancer, cardiovascular diseases were found to account for a considerable proportion of deaths in long-term lung cancer survivors [19, 20]. Another study on NSCLC indicating cardiovascular diseases accounted for approximately 5.3% of the total death, only secondary to primary cancer [21]; similar conclusions were reached in our study, where cardiovascular diseases were found to be a formidable health problem in SCLC, especially in patients survived longer than three years. Studies have demonstrated that the elevated incidence of cardiovascular diseases may be partly due to toxicities of cancer treatment including radiotherapy and chemotherapy [17]. Recently, with the development of immunotherapy in SCLC, immune-related cardiovascular diseases are increasingly recognized, and this will further increase the incidence of cardiovascular death [22]. Thus, the early involvement of cardiologists in such patients is recommended to provide optimal comprehensive care.

COPD was another common cause of non-cancer CODs in our analysis during all examined latency periods and in all subgroups. As a systemic inflammatory disease, COPD has been proved to be associated with many systemic comorbidities [23, 24]. Recently, studies found that chronic inflammation in COPD could also promote tumorigenesis through inducing the expression of STAT3 and other growth factors [25]. A higher prevalence of COPD was reported in lung cancer patients and vice versa, its presence was also found to be closely related to the prognosis of SCLC [26, 27]. Moreover, the incidence of vascular-related events was also higher in COPD patients, which may further undermine the prognosis of patients with SCLC [28]. Such tight association between COPD, cardiovascular events and SCLC may be partly attributed to shared risk factors like tobacco use and age [14, 29]. Thus, the smoking cessation campaign is called in this population.

In addition to cardiopulmonary death, the risk of death from septicemia was also increased in SCLC, especially in patients diagnosed with SCLC within one year. It may be explained by the fact that septicemia in SCLC is often associated with tumor-related treatments, including surgery, chemotherapy, and radiotherapy [30]. Studies reported that sepsis after surgery or chemotherapy could lead to a decline in cancer survival rates [31, 32]. Therefore, the monitoring of infection-related indicators is also an important part of treatment strategies for SCLC.

Finally, given the fact that the risk of suicide significantly increases within the first years after SCLC diagnosis, psychiatric assessment and support should also be incorporated into initial treatment plans [33–35].

Like other retrospective studies, the current study had several limitations that should be acknowledged. First, due to the inherent weaknesses of the SEER database, we do not have detailed information on disease recurrence, postoperative complications and treatments, one or all of which will influence the survival durations captured in this work and may complicate the interpretation of survival and death patterns. However, these limitations apply to all population-based analyses based on SEER or other similar large-scale data repositories. Second, for patients with more than one fatal complication, some CODs may be underreported, leading to potential bias. Finally, due to the large sample size, it is possible that some statistically significant findings are accidental and not necessarily clinically significant, thus, it is important to interpret these results through the absolute value of SMR. Despite these limitations, our work is the first to provide CODs distribution in SCLC, which may provide new insights into the treatment and health risk counseling.

## Conclusion

Patients with SCLC were at a higher risk of dying from most non-cancer CODs. Furthermore, during follow-up after SCLC diagnosis, the incidence of death from non-SCLC causes increased as survival time was prolonged. Cardiovascular events, COPD and septicemia were the most common causes. These findings highlight the importance of multidisciplinary care for the follow-up strategy in SCLC.

## Supplementary Information

**Additional file 1.** Definition of each cause of death and corresponding codes in the ICD-10.

**Additional file 2.** SMRs for each cause of death following SCLC diagnosis in patients younger than 60 years.

**Additional file 3.** SMRs for each cause of death following SCLC diagnosis in patients aged 60-69 years.

**Additional file 4.** SMRs for each cause of death following SCLC diagnosis in patients aged more than 70 years.

**Additional file 5.** SMRs for each cause of death following SCLC diagnosis in white patients.

**Additional file 6.** SMRs for each cause of death following SCLC diagnosis in black patients.

**Additional file 7.** SMRs for each cause of death following SCLC diagnosis in patients of other races.

**Additional file 8.** SMRs for each cause of death following SCLC diagnosis in patients undergoing chemotherapy.

**Additional file 9.** SMRs for each cause of death following SCLC diagnosis in patients undergoing radiotherapy.

**Additional file 10.** SMRs for each cause of death in patients with stage I-III SCLC.

**Additional file 11.** SMRs for each cause of death in patients with stage IV SCLC.

## Data Availability

The original data came from the SEER database. All data discussed in the manuscript are included within this published article.

## References

[1] Chauhan AF, Liu SV (2020). Small cell lung cancer: advances in diagnosis and management. Semin Respir Crit Care Med.

[2] Wang Y, Zou S, Zhao Z, Liu P, Ke C, Xu S (2020). New insights into small-cell lung cancer development and therapy. Cell Biol Int.

[3] Kalemkerian GP, Schneider BJ (2017). Advances in small cell lung cancer. Hematol Oncol Clin North Am.

[4] Pandey M, Mukhopadhyay A, Sharawat SK, Kumar S (2021). Role of microRNAs in regulating cell proliferation, metastasis and chemoresistance and their applications as cancer biomarkers in small cell lung cancer. Biochim Biophys Acta Rev Cancer.

[5] Glatzer M, Rittmeyer A, Müller J (2017). Treatment of limited disease small cell lung cancer: the multidisciplinary team. Eur Respir J..

[6] Sabari JK, Lok BH, Laird JH, Poirier JT, Rudin CM (2017). Unravelling the biology of SCLC: implications for therapy. Nat Rev Clin Oncol.

[7] Tian Y, Zhai X, Han A, Zhu H, Yu J (2019). Potential immune escape mechanisms underlying the distinct clinical outcome of immune checkpoint blockades in small cell lung cancer. J Hematol Oncol.

[8] Xu Y, Zhan P, Song Y (2020). Immunotherapy advances in small cell lung cancer. Zhongguo Fei Ai Za Zhi..

[9] Baxi SS, Pinheiro LC, Patil SM, Pfister DG, Oeffinger KC, Elkin EB (2014). Causes of death in long-term survivors of head and neck cancer. Cancer.

[10] Bergmann MM, Rehm J, Klipstein-Grobusch K (2013). The association of pattern of lifetime alcohol use and cause of death in the European prospective investigation into cancer and nutrition (EPIC) study. Int J Epidemiol.

[11] Horn SR, Stoltzfus KC, Mackley HB (2020). Long-term causes of death among pediatric patients with cancer. Cancer.

[12] Xie SH, Chen H, Lagergren J (2020). Causes of death in patients diagnosed with gastric adenocarcinoma in Sweden, 1970–2014: a population-based study. Cancer Sci.

[13] Bray F, Ferlay J, Soerjomataram I, Siegel RL, Torre LA, Jemal A (2018). Global cancer statistics 2018: GLOBOCAN estimates of incidence and mortality worldwide for 36 cancers in 185 countries. CA Cancer J Clin.

[14] McRobbie H, Kwan B (2021). Tobacco use disorder and the lungs. Addiction.

[15] Chen RC, Royce TJ, Extermann M, Reeve BB (2012). Impact of age and comorbidity on treatment and outcomes in elderly cancer patients. Semin Radiat Oncol.

[16] Kim MG, Kim HS, Kim BS, Kwon SJ (2013). The impact of old age on surgical outcomes of totally laparoscopic gastrectomy for gastric cancer. Surg Endosc.

[17] Ruparel M, Quaife SL, Dickson JL (2019). Evaluation of cardiovascular risk in a lung cancer screening cohort. Thorax.

[18] Sun JY, Zhang ZY, Qu Q (2021). Cardiovascular disease-specific mortality in 270,618 patients with non-small cell lung cancer. Int J Cardiol.

[19] Abdel-Rahman O (2017). Causes of death in long-term lung cancer survivors: a SEER database analysis. Curr Med Res Opin.

[20] Zaorsky NG, Churilla TM, Egleston BL (2017). Causes of death among cancer patients. Ann Oncol.

[21] Hubbard MO, Fu P, Margevicius S, Dowlati A, Linden PA (2012). Five-year survival does not equal cure in non-small cell lung cancer: a surveillance, epidemiology, and end results-based analysis of variables affecting 10- to 18-year survival. J Thorac Cardiovasc Surg.

[22] Salem JE, Manouchehri A, Moey M (2018). Cardiovascular toxicities associated with immune checkpoint inhibitors: an observational, retrospective, pharmacovigilance study. Lancet Oncol.

[23] Mouronte-Roibás C, Leiro-Fernández V, Fernández-Villar A, Botana-Rial M, Ramos-Hernández C, Ruano-Ravina A (2016). COPD, emphysema and the onset of lung cancer: a systematic review. Cancer Lett.

[24] Bozinovski S, Vlahos R, Anthony D (2016). COPD and squamous cell lung cancer: aberrant inflammation and immunity is the common link. Br J Pharmacol.

[25] Qu P, Roberts J, Li Y (2009). Stat3 downstream genes serve as biomarkers in human lung carcinomas and chronic obstructive pulmonary disease. Lung Cancer.

[26] Houghton AM (2013). Mechanistic links between COPD and lung cancer. Nat Rev Cancer.

[27] Liao KM, Hung CM, Shu CC, Lee HS, Wei YF (2021). Impact of chronic obstructive pulmonary disease on the mortality of patients with small cell lung cancer. Int J Chron Obstruct Pulmon Dis.

[28] Cavaillès A, Brinchault-Rabin G, Dixmier A (2013). Comorbidities of COPD. Eur Respir Rev.

[29] Tønnesen P (2013). Smoking cessation and COPD. Eur Respir Rev.

[30] Mirouse A, Vigneron C, Llitjos JF (2020). Sepsis and cancer: an interplay of friends and foes. Am J Respir Crit Care Med.

[31] Hiong A, Thursky KA, Teh BW, Haeusler GM, Slavin MA, Worth LJ (2016). Sepsis following cancer surgery: the need for early recognition and standardised clinical care. Expert Rev Anti Infect Ther.

[32] Liu Z, Mahale P, Engels EA (2019). Sepsis and risk of cancer among elderly adults in the United States. Clin Infect Dis.

[33] Amiri S, Behnezhad S (2020). Cancer diagnosis and suicide mortality: a systematic review and meta-analysis. Arch Suicide Res.

[34] Guo Z, Gu C, Li S (2021). Incidence and risk factors of suicide among patients diagnosed with bladder cancer: a systematic review and meta-analysis. Urol Oncol.

[35] Zaorsky NG, Zhang Y, Tuanquin L, Bluethmann SM, Park HS, Chinchilli VM (2019). Suicide among cancer patients. Nat Commun.

