# Supplementary material for: Causes of death following small cell lung cancer diagnosis: a population-based analysis
Source: BMC Pulm Med. 2022 Jul 4;22:262. doi: 10.1186/s12890-022-02053-4 (PMC9254402; doi:10.1186/s12890-022-02053-4)
Supplement: Supplementary file 1 — Additional file 1. Definition of each cause of death and corresponding codes in the ICD-10. [file 12890_2022_2053_MOESM1_ESM.docx]

Supplemental Table 1. Definition of each cause of death and corresponding codes in the ICD-10

| **Non-Cancer Causes of Death** | **ICD-10 corresponding codes** | **Cause of death definition** |
| --- | --- | --- |
| Septicemia | A40-A41 | Sepsis |
| Infectious/parasitic diseases | A00-A08 | Intestinal infectious diseases |
|  | A15-A19 | Tuberculosis |
|  | A20-A33 | Certain zoonotic bacterial diseases |
|  | A35-A39 | Other bacterial diseases: tetanus, diphtheria, whooping cough, Scarlet fever and meningococcal infection |
|  | A42-A49 | Other bacterial diseases: actinomycosis, nocardiosis, bartonellosis, erysipelas and other bacterial infections of unspecified site |
|  | A50-A53 | Syphilis |
|  | A54-B19 | Infections with a predominantly sexual mode of transmission, other spirochaetal diseases, other diseases caused by chlamydiae, rickettsioses, viral infections of the central nervous system, arthropod-borne viral fevers and viral haemorrhagic fevers, viral infections characterized by skin and mucous membrane lesions and viral hepatitis |
|  | B25-B99 | Other viral diseases, mycoses, protozoal diseases, helminthiases, pediculosis, acariasis and other infestations, sequelae of infectious and parasitic diseases, bacterial, viral and other infectious agents and diseases |
| Diabetes mellitus | E10-E14 | Diabetes mellitus |
| Alzheimer’s disease | G30 | Alzheimer’s disease |
| Cardiovascular Diseases | I00-I02 | Acute rheumatic fever |
|  | I05-I09 | Chronic rheumatic heart diseases |
|  | I11 | Hypertensive heart disease |
|  | I13 | Hypertensive heart and renal disease |
|  | I20-I25 | Ischemic heart diseases |
|  | I26-I28 | Pulmonary heart disease and diseases of pulmonary circulation |
|  | I30-I32 | Diseases of pericardium |
|  | I33 | Acute and subacute endocarditis |
|  | I34-I39 | Nonrheumatic valve disorders |
|  | I40-I41 | Myocarditis |
|  | I42-I43 | Cardiomyopathy |
|  | I44-I45 | Conduction disorders |
|  | I46 | Cardiac arrest |
|  | I47-I49 | Arrythmias |
|  | I50 | Heart failure |
|  | I51 | Complications and ill-defined descriptions of heart disease |
|  | I10 | Essential (primary) hypertension |
|  | I12 | Hypertensive renal disease |
|  | I70 | Atherosclerosis |
|  | I71 | Aortic Aneurysm and Dissection |
|  | I72-I73 | Other aneurysm and dissection or other peripheral vascular diseases |
|  | I74 | Arterial embolism and thrombosis |
|  | I77 | Other disorders of arteries and arterioles |
|  | I78 | Diseases of capillaries |
| Cerebrovascular Diseases | I60-I62 | Nontraumatic intracranial haemorrhage |
|  | I63 | Cerebral infarction |
|  | I64 | Stroke, not specified as haemorrhage or infarction |
|  | I65-I66 | Occlusion and stenosis of precerebral/cerebral arteries, not resulting in cerebral infarction |
|  | I67-I69 | Other cerebrovascular diseases or Sequelae of cerebrovascular disease |
| Pneumonia and Influenza | J09-J18 | Influenza and pneumonia |
| Chronic Obstructive Pulmonary Disease | J40-J42 | Bronchitis |
|  | J43 | Emphysema |
|  | J44 | Other chronic obstructive pulmonary disease |
|  | J45-J46 | Asthma or Status asthmaticus |
|  | J47 | Bronchiectasis |
| Chronic Liver Disease and Cirrhosis | K70 | Alcoholic liver disease |
|  | K73 | Chronic hepatitis |
|  | K74 | Fibrosis and cirrhosis of liver |
| Nephritis, Nephrotic Syndrome and Nephrosis | N00-N07 | Glomerular diseases |
|  | N17-N19 | Renal failure |
|  | N25 | Disorders resulting from impaired renal tubular function |
|  | N26 | Unspecified contracted kidney |
|  | N27 | Small kidney of unknown cause |
| Accidents and Adverse Effects | V01-V99 | Transport accidents |
|  | W00-X59 | Other external causes of accidental injury |
|  | Y85-Y86 | Sequelae of transport accidents or other accidents |
| Suicide and Self-Inflicted Injury | X60-X84 | Intentional self-harm |
|  | Y87 | Sequelae of intentional self-harm, assault and events of undetermined intent |
| Other Cause of Death | D00-D09 | In situ neoplasms |
|  | D10-D36 | Benign neoplasms |
|  | D37-D48 | Neoplasms of uncertain or unknown behavior |
|  | K25-K28 | Stomach and Duodenal Ulcers |
|  | A34 | Complications of Pregnancy, Childbirth, Puerperium: Obstetrical tetanus |
|  | O00-O95 | Complications of Pregnancy, Childbirth, Puerperium: Pregnancy with abortive outcome, oedema, proteinuria and hypertensive disorders in pregnancy, childbirth and the puerperium, other maternal disorders predominantly related to pregnancy, maternal care related to the fetus and amniotic cavity and possible delivery problems and other complications of labour, delivery and the puerperium |
|  | O98-O99 | Complications of Pregnancy, Childbirth, Puerperium: Maternal infectious, parasitic and other diseases |
|  | Q00-Q99 | Congenital Anomalies |
|  | R00-R99 | Symptoms, signs, abnormal results of clinical or other investigative procedures, and ill-defined conditions regarding which no diagnosis classifiable elsewhere is recorded. |
|  | P00-P96 | Certain Conditions Originating in Perinatal Period: Fetus and newborn affected by maternal factors and by complications of pregnancy, labour and delivery, disorders related to length of gestation and fetal growth, birth trauma, respiratory and cardiovascular disorders specific to the perinatal period, infections specific to the perinatal period haemorrhagic and haematological disorders of fetus and newborn, transitory endocrine and metabolic disorders specific to fetus and newborn, digestive system disorders of fetus and newborn, conditions involving the integument and temperature regulation of fetus and newborn and other disorders originating in the perinatal period |
|  | X85-Y09 | Homicide and Legal Intervention: Assault |
|  | Y35 | Legal Intervention |
|  | Y87 | Sequelae of assault |
|  | Y89 | Sequelae of legal intervention, war operations and unspecified external cause |
