# Supplementary material for: Causes of death following small cell lung cancer diagnosis: a population-based analysis
Source: BMC Pulm Med. 2022 Jul 4;22:262. doi: 10.1186/s12890-022-02053-4 (PMC9254402; doi:10.1186/s12890-022-02053-4)
Supplement: Supplementary file 2 — Additional file 2. SMRs for each cause of death following SCLC diagnosis in patients younger than 60 years. [file 12890_2022_2053_MOESM2_ESM.docx]

|  | Deaths by time after diagnosis | | | | | |  | |
| --- | --- | --- | --- | --- | --- | --- | --- | --- |
|  | <1 y | | 1-3 y | | >3 y | | Total deaths | |
|  | Observed,  No. | SMR (95% CI) | Observed,  No. | SMR (95% CI) | Observed,  No. | SMR (95% CI) | Observed,  No. | SMR (95% CI) |
| Cause of death |  |  |  |  |  |  |  |  |
| All | 6 434 | 138.3 (135.0-141.8)^*^ | 3 558 | 121.2 (117.2-125.2) ^*^ | 574 | 14.86 (13.67-16.13) ^*^ | 10 566 | 92.31 (90.55-94.08)^*^ |
| SCLC | 5 811 | 1499 (1461-1538) ^*^ | 3 308 | 1,302 (1258-1347) ^*^ | 428 | 123.1(111.8-135.4) ^*^ | 9 547 | 965.4 (946.2-984.9) ^*^ |
| Other cancers | 315 | 29.78 (26.59-33.26) ^*^ | 120 | 17.30 (14.34-20.68) ^*^ | 19 | 2.05 (1.23-3.20) ^*^ | 454 | 16.95 (15.43-18.59) ^*^ |
| Noncancer causes |  |  |  |  |  |  |  |  |
| Septicemia | 20 | 30.04 (18.35-46.40) ^*^ | 8 | 17.95 (7.75-35.38) ^*^ | 5 | 7.92 (2.57-18.48) ^*^ | 33 | 18.94 (13.04-26.60) ^*^ |
| Infectious/ parasitic diseases  including HIV infection | 22 | 17.29 (10.83-26.17) ^*^ | 1 | 1.45 (0.04-8.10) | 2 | 3.01 (0.36-10.86) | 25 | 9.52 (6.16-14.05) ^*^ |
| Diabetes mellitus | 3 | 1.8 (0.37-5.27) | 3 | 2.76 (0.57-8.08) | 4 | 2.67 (0.73-6.83) | 10 | 2.36 (1.13-4.33) ^*^ |
| Alzheimer’s disease | 0 | 0 (0.00-57.94) | 0 | 0 (0.00-63.95) | 0 | 0 (0.00-21.21) | 0 | 0 (0.00-12.49) |
| Cardiovascular diseases | 72 | 6.74 (5.27-8.49) ^*^ | 47 | 7.12 (5.23-9.47) ^*^ | 34 | 3.88 (2.68-5.42) ^*^ | 153 | 5.87 (4.98-6.88) ^*^ |
| Cerebrovascular diseases | 11 | 7.11 (3.55-12.73) ^*^ | 6 | 6.01 (2.21-13.08) ^*^ | 7 | 5.08 (2.04-10.46) ^*^ | 24 | 6.12 (3.92-9.10) ^*^ |
| Pneumonia and influenza | 15 | 26.04 (14.57-42.94) ^*^ | 3 | 7.92 (1.63-23.14) ^*^ | 4 | 7.31 (1.99-18.71) ^*^ | 22 | 14.65 (9.18-22.17) ^*^ |
| COPD/ associated conditions | 20 | 12.23 (7.47-18.88) ^*^ | 13 | 10.82 (5.76-18.50) ^*^ | 28 | 13.52 (8.98-19.53) ^*^ | 61 | 12.43 (9.51-15.96) ^*^ |
| Chronic liver disease/ cirrhosis | 4 | 2.19 (0.60-5.61) | 1 | 0.94 (0.02-5.21) | 5 | 4.17 (1.36-9.74) ^*^ | 10 | 2.44 (1.17-4.50) ^*^ |
| Nephritis, nephrotic syndrome, and nephrosis | 9 | 14.33 (6.55-27.20) ^*^ | 5 | 11.83 (3.84-27.61) ^*^ | 2 | 3.21(0.39-11.60) | 16 | 9.56 (5.46-15.52) ^*^ |
| Accidents and adverse effects of medications | 24 | 6.97 (4.47-10.38) ^*^ | 7 | 3.57 (1.43-7.35) ^*^ | 11 | 5.53 (2.76-9.90) ^*^ | 42 | 5.68 (4.10-7.68) ^*^ |
| Suicide and self-inflicted injury | 5 | 3.51 (1.14-8.19) ^*^ | 3 | 3.88 (0.80-11.33) | 1 | 1.35(0.03-7.54) | 9 | 3.06 (1.40-5.82) ^*^ |
| Other | 103 | 15.57 (12.71-18.88) ^*^ | 33 | 7.88 (5.42-11.06) ^*^ | 24 | 4.29 (2.75-6.38) ^*^ | 160 | 9.75 (8.30-11.39) ^*^ |

Supplementary Table 2. SMRs for each cause of death following SCLC diagnosis in patients younger than 60 years

* indicated p<0.05.
