# Supplementary material for: Causes of death following small cell lung cancer diagnosis: a population-based analysis
Source: BMC Pulm Med. 2022 Jul 4;22:262. doi: 10.1186/s12890-022-02053-4 (PMC9254402; doi:10.1186/s12890-022-02053-4)
Supplement: Supplementary file 3 — Additional file 3. SMRs for each cause of death following SCLC diagnosis in patients aged 60-69 years. [file 12890_2022_2053_MOESM3_ESM.docx]

Supplementary Table 3. SMRs for each cause of death following SCLC diagnosis in patients aged 60-69 years

|  | Deaths by time after diagnosis | | | | | |  | |
| --- | --- | --- | --- | --- | --- | --- | --- | --- |
|  | <1 y | | 1-3 y | | >3 y | | Total deaths | |
|  | Observed,  No. | SMR (95% CI) | Observed,  No. | SMR (95% CI) | Observed,  No. | SMR (95% CI) | Observed,  No. | SMR (95% CI) |
| Cause of death |  |  |  |  |  |  |  |  |
| All | 9 873 | 76.32 (74.82-77.84)^*^ | 4 129 | 56.82 (55.10-58.58) ^*^ | 729 | 9.71 (9.02-10.44) ^*^ | 14 731 | 53.16 (52.30-54.02) ^*^ |
| SCLC | 8 827 | 594.9 (582.5-607.4) ^*^ | 3 787 | 473.2 (458.3-488.5) ^*^ | 480 | 64.96 (59.28-71.04) ^*^ | 13 094 | 433.1 (425.7-440.6) ^*^ |
| Other cancers | 424 | 13.52 (12.27-14.87) ^*^ | 137 | 7.86 (6.60-9.29) ^*^ | 19 | 1.14 (0.68-1.77) | 580 | 8.85 (8.15-9.61) ^*^ |
| Noncancer causes |  |  |  |  |  |  |  |  |
| Septicemia | 40 | 19.47 (13.91-26.51) ^*^ | 10 | 8.36 (4.01-15.38) ^*^ | 5 | 3.93 (1.28-9.18) ^*^ | 55 | 12.17 (9.16-15.83) ^*^ |
| Infectious/ parasitic diseases  including HIV infection | 19 | 12.36 (7.44-19.30) ^*^ | 1 | 1.25 (0.03-6.95) | 5 | 7.42 (2.41-17.32) ^*^ | 25 | 8.30 (5.37-12.25) ^*^ |
| Diabetes mellitus | 14 | 2.73 (1.49-4.58) ^*^ | 2 | 0.7 (0.08-2.53) | 4 | 1.43 (0.39-3.66) | 20 | 1.86 (1.13-2.87) ^*^ |
| Alzheimer’s disease | 2 | 2.67 (0.32-9.64) | 0 | 0 (0.00-6.16) | 12 | 8.92 (4.61-15.58) ^*^ | 14 | 5.20 (2.84-8.72) ^*^ |
| Cardiovascular diseases | 185 | 5.83 (5.02-6.73) ^*^ | 67 | 3.87 (3.00-4.91) ^*^ | 46 | 2.60 (1.91-3.47) ^*^ | 298 | 4.47 (3.97-5.00) ^*^ |
| Cerebrovascular diseases | 23 | 4.54 (2.88-6.82) ^*^ | 10 | 3.38 (1.62-6.22) ^*^ | 23 | 6.66 (4.22-10.00) ^*^ | 56 | 4.88 (3.69-6.34) ^*^ |
| Pneumonia and influenza | 24 | 13.15 (8.43-19.57) ^*^ | 6 | 5.50 (2.02-11.98) ^*^ | 5 | 3.90 (1.27-9.10) ^*^ | 35 | 8.34 (5.81-11.60) ^*^ |
| COPD/ associated conditions | 95 | 11.34 (9.18-13.87) ^*^ | 26 | 5.09 (3.33-7.46) ^*^ | 57 | 9.53 (7.22-12.35) ^*^ | 178 | 9.15 (7.85-10.59) ^*^ |
| Chronic liver disease/ cirrhosis | 8 | 3.05 (1.32-6.02) ^*^ | 3 | 2.22 (0.46-6.49) | 1 | 0.94 (0.02-5.24) | 12 | 2.38 (1.23-4.16) ^*^ |
| Nephritis, nephrotic syndrome, and nephrosis | 6 | 2.65 (0.97-5.77) | 4 | 3 (0.82-7.68) | 3 | 2.02 (0.42-5.91) | 13 | 2.56 (1.36-4.37) ^*^ |
| Accidents and adverse effects of medications | 19 | 5.01 (3.02-7.83) ^*^ | 11 | 5.41 (2.70-9.68) ^*^ | 16 | 8.55 (4.89-13.89) ^*^ | 46 | 5.98 (4.38-7.97) ^*^ |
| Suicide and self-inflicted injury | 11 | 7.88 (3.93-14.09) ^*^ | 0 | 0 (0.00-5.36) | 0 | 0 (0.00-7.18) | 11 | 4.23 (2.11-7.57) ^*^ |
| Other | 176 | 10.57 (9.07-12.26) ^*^ | 65 | 6.56 (5.06-8.36) ^*^ | 53 | 4.58 (3.43-5.98) ^*^ | 294 | 7.71 (6.85-8.64) ^*^ |

* indicated p<0.05.
