# Supplementary material for: Causes of death following small cell lung cancer diagnosis: a population-based analysis
Source: BMC Pulm Med. 2022 Jul 4;22:262. doi: 10.1186/s12890-022-02053-4 (PMC9254402; doi:10.1186/s12890-022-02053-4)
Supplement: Supplementary file 4 — Additional file 4. SMRs for each cause of death following SCLC diagnosis in patients aged more than 70 years. [file 12890_2022_2053_MOESM4_ESM.docx]

Supplementary Table 4. SMRs for each cause of death following SCLC diagnosis in patients aged more than 70 years

|  | Deaths by time after diagnosis | | | | | |  | |
| --- | --- | --- | --- | --- | --- | --- | --- | --- |
|  | <1 y | | 1-3 y | | >3 y | | Total deaths | |
|  | Observed,  No. | SMR (95% CI) | Observed,  No. | SMR (95% CI) | Observed,  No. | SMR (95% CI) | Observed,  No. | SMR (95% CI) |
| Cause of death |  |  |  |  |  |  |  |  |
| All | 13 229 | 36.59 (35.97-37.22)^*^ | 3 353 | 21.67 (20.94-22.42) ^*^ | 597 | 5.05 (4.65-5.47) ^*^ | 17 179 | 27.07 (26.67-27.48) ^*^ |
| SCLC | 11 668 | 442.4 (434.4-450.5) ^*^ | 3 020 | 281.8 (271.8-292.0) ^*^ | 350 | 56.09 (50.37-62.29) ^*^ | 15 038 | 347.0 (341.5-352.6) ^*^ |
| Other cancers | 570 | 9.11 (8.38-9.89) ^*^ | 84 | 3.20 (2.56-3.97) ^*^ | 19 | 1.09 (0.66-1.70) | 673 | 6.34 (5.87-6.83) ^*^ |
| Noncancer causes |  |  |  |  |  |  |  |  |
| Septicemia | 46 | 8.24 (6.03-10.99) ^*^ | 11 | 4.55 (2.27-8.14) ^*^ | 7 | 3.91 (1.57-8.05) ^*^ | 64 | 6.54 (5.03-8.35) ^*^ |
| Infectious/ parasitic diseases  including HIV infection | 15 | 5.97 (3.34-9.84) ^*^ | 2 | 1.83 (0.22-6.62) | 2 | 2.63 (0.32-9.49) | 19 | 4.35 (2.62-6.79) ^*^ |
| Diabetes mellitus | 17 | 1.51 (0.88-2.42) | 1 | 0.21 (0.01-1.18) | 1 | 0.31 (0.01-1.73) | 19 | 0.99 (0.60-1.55) |
| Alzheimer’s disease | 5 | 0.38 (0.12-0.89) ^*^ | 4 | 0.63 (0.17-1.61) | 14 | 2.10 (1.15-3.52) ^*^ | 23 | 0.88 (0.56-1.32) |
| Cardiovascular diseases | 336 | 3.28 (2.94-3.65) ^*^ | 86 | 2.01 (1.61-2.48) ^*^ | 48 | 1.44 (1.06-1.91) ^*^ | 470 | 2.63 (2.40-2.88) ^*^ |
| Cerebrovascular diseases | 41 | 1.89 (1.36-2.57) ^*^ | 13 | 1.39 (0.74-2.38) | 15 | 1.99 (1.11-3.28) ^*^ | 69 | 1.79 (1.39-2.27) ^*^ |
| Pneumonia and influenza | 37 | 4.29 (3.02-5.91) ^*^ | 6 | 1.65 (0.61-3.60) | 13 | 4.49 (2.39-7.67) ^*^ | 56 | 3.69 (2.79-4.80) ^*^ |
| COPD/ associated conditions | 177 | 6.79 (5.82-7.86) ^*^ | 47 | 4.19 (3.08-5.58) ^*^ | 66 | 8.20 (6.34-10.43) ^*^ | 290 | 6.40 (5.68-7.18) ^*^ |
| Chronic liver disease/ cirrhosis | 3 | 1.24 (0.26-3.63) | 0 | 0 (0.00-3.66) | 0 | 0 (0.00-6.30) | 3 | 0.75 (0.15-2.19) |
| Nephritis, nephrotic syndrome, and nephrosis | 16 | 2.05 (1.17-3.32) ^*^ | 3 | 0.89 (0.18-2.61) | 6 | 2.36 (0.87-5.14) | 25 | 1.82 (1.18-2.69) ^*^ |
| Accidents and adverse effects of medications | 27 | 3.34 (2.20-4.85) ^*^ | 11 | 3.12 (1.56-5.57) ^*^ | 7 | 2.46 (0.99-5.06) | 45 | 3.11 (2.27-4.16) ^*^ |
| Suicide and self-inflicted injury | 7 | 5.00 (2.01-10.30) ^*^ | 5 | 9.16 (2.97-21.38) ^*^ | 1 | 3.06 (0.08-17.03) | 13 | 5.72 (3.04-9.78) ^*^ |
| Other | 264 | 4.28 (3.78-4.83) ^*^ | 60 | 2.16 (1.65-2.78) ^*^ | 48 | 2.00 (1.47-2.65) ^*^ | 372 | 3.28 (2.95-3.63) ^*^ |

* indicated p<0.05.
