# Supplementary material for: Causes of death following small cell lung cancer diagnosis: a population-based analysis
Source: BMC Pulm Med. 2022 Jul 4;22:262. doi: 10.1186/s12890-022-02053-4 (PMC9254402; doi:10.1186/s12890-022-02053-4)
Supplement: Supplementary file 5 — Additional file 5. SMRs for each cause of death following SCLC diagnosis in white patients. [file 12890_2022_2053_MOESM5_ESM.docx]

Supplementary Table 5. SMRs for each cause of death following SCLC diagnosis in white patients

|  | Deaths by time after diagnosis | | | | | |  | |
| --- | --- | --- | --- | --- | --- | --- | --- | --- |
|  | <1 y | | 1-3 y | | >3 y | | Total deaths | |
|  | Observed,  No. | SMR (95% CI) | Observed,  No. | SMR (95% CI) | Observed,  No. | SMR (95% CI) | Observed,  No. | SMR (95% CI) |
| Cause of death |  |  |  |  |  |  |  |  |
| All | 25 603 | 55.75(55.07-56.44) ^*^ | 9 458 | 43.36(42.49-44.24) ^*^ | 1 623 | 8.21(7.82-8.62) ^*^ | 36 684 | 41.93(41.50-42.36) ^*^ |
| SCLC | 22 886 | 583.9(576.3-591.5) ^*^ | 8 673 | 471.2(461.3-481.2) ^*^ | 1 065 | 71.42(67.20-75.84) ^*^ | 32 624 | 449.9(445.0-454.8) ^*^ |
| Other cancers | 1 113 | 12.49(11.77-13.25) ^*^ | 288 | 6.72(5.96-7.54) ^*^ | 50 | 1.35(1.00-1.78) ^*^ | 1 451 | 8.59(8.15-9.04) ^*^ |
| Noncancer causes |  |  |  |  |  |  |  |  |
| Septicemia | 89 | 13.38(10.74-16.46) ^*^ | 20 | 6.17(3.77-9.53) ^*^ | 14 | 4.69(2.57-7.88) ^*^ | 123 | 9.55(7.94-11.40) ^*^ |
| Infectious/ parasitic diseases  including HIV infection | 38 | 9.27(6.56-12.72) ^*^ | 3 | 1.51(0.31-4.42) | 7 | 4.21(1.69-8.67) ^*^ | 48 | 6.20(4.57-8.22) ^*^ |
| Diabetes mellitus | 26 | 1.84(1.20-2.70) ^*^ | 5 | 0.74(0.24-1.73) | 6 | 1.02(0.37-2.22) | 37 | 1.38(0.97-1.91) |
| Alzheimer’s disease | 5 | 0.39(0.13-0.92) ^*^ | 4 | 0.63(0.17-1.61) | 24 | 3.30(2.11-4.91) ^*^ | 33 | 1.25(0.86-1.76) |
| Cardiovascular diseases | 472 | 3.86(3.52-4.23) ^*^ | 170 | 3.05(2.61-3.54) ^*^ | 115 | 2.30(1.90-2.76) ^*^ | 757 | 3.32(3.09-3.57) ^*^ |
| Cerebrovascular diseases | 64 | 2.75(2.12-3.51) ^*^ | 25 | 2.31(1.49-3.40) ^*^ | 35 | 3.44(2.40-4.79) ^*^ | 124 | 2.80(2.33-3.34) ^*^ |
| Pneumonia and influenza | 60 | 6.41(4.89-8.25) ^*^ | 12 | 2.79(1.44-4.87) ^*^ | 18 | 4.53(2.69-7.16) ^*^ | 90 | 5.10(4.10-6.27) ^*^ |
| COPD/ associated conditions | 265 | 8.01(7.07-9.03) ^*^ | 80 | 4.99(3.96-6.21) ^*^ | 138 | 9.36(7.87-11.06) ^*^ | 483 | 7.56(6.90-8.27) ^*^ |
| Chronic liver disease/ cirrhosis | 14 | 2.29(1.25-3.85) ^*^ | 3 | 0.99(0.20-2.89) | 4 | 1.57(0.43-4.02) | 21 | 1.80(1.11-2.75) ^*^ |
| Nephritis, nephrotic syndrome, and nephrosis | 26 | 3.13(2.04-4.58) ^*^ | 11 | 2.80(1.40-5.02) ^*^ | 7 | 1.95(0.78-4.02) | 44 | 2.78(2.02-3.73) ^*^ |
| Accidents and adverse effects of medications | 61 | 4.56(3.49-5.86) ^*^ | 27 | 4.13(2.72-6.01) ^*^ | 29 | 4.95(3.31-7.10) ^*^ | 117 | 4.54(3.75-5.44) ^*^ |
| Suicide and self-inflicted injury | 22 | 5.51(3.46-8.35) ^*^ | 7 | 3.70(1.49-7.62) ^*^ | 2 | 1.34(0.16-4.84) | 31 | 4.20(2.86-5.97) ^*^ |
| Other | 462 | 6.27(5.71-6.87) ^*^ | 130 | 3.59(3.00-4.27) ^*^ | 109 | 3.06(2.51-3.69) ^*^ | 701 | 4.82(4.47-5.19) ^*^ |

* indicated p<0.05.
