# Supplementary material for: Causes of death following small cell lung cancer diagnosis: a population-based analysis
Source: BMC Pulm Med. 2022 Jul 4;22:262. doi: 10.1186/s12890-022-02053-4 (PMC9254402; doi:10.1186/s12890-022-02053-4)
Supplement: Supplementary file 6 — Additional file 6. SMRs for each cause of death following SCLC diagnosis in black patients. [file 12890_2022_2053_MOESM6_ESM.docx]

|  | Deaths by time after diagnosis | | | | | |  | |
| --- | --- | --- | --- | --- | --- | --- | --- | --- |
|  | <1 y | | 1-3 y | | >3 y | | Total deaths | |
|  | Observed,  No. | SMR (95% CI) | Observed,  No. | SMR (95% CI) | Observed,  No. | SMR (95% CI) | Observed,  No. | SMR (95% CI) |
| Cause of death |  |  |  |  |  |  |  |  |
| All | 2 920 | 46.71(45.03-48.44) ^*^ | 1 180 | 37.84(35.71-40.06) ^*^ | 206 | 7.42(6.44-8.51) ^*^ | 4 306 | 35.46(34.41-36.53) ^*^ |
| SCLC | 2 522 | 531.9(511.3-553.1) ^*^ | 1 069 | 463.7(436.3-492.4) ^*^ | 148 | 85.09(71.93-99.95) ^*^ | 3 739 | 425.5(412.0-439.4) ^*^ |
| Other cancers | 155 | 12.58(10.68-14.72) ^*^ | 45 | 7.29(5.31-9.75) ^*^ | 4 | 0.78(0.21-1.99) | 204 | 8.63(7.49-9.90) ^*^ |
| Noncancer causes |  |  |  |  |  |  |  |  |
| Septicemia | 12 | 8.35(4.31-14.58) ^*^ | 7 | 9.80(3.94-20.18) ^*^ | 2 | 3.22(0.39-11.62) | 21 | 7.57(4.69-11.57) ^*^ |
| Infectious/ parasitic diseases  including HIV infection | 16 | 15.18(8.68-24.66) ^*^ | 1 | 1.94(0.05-10.78) | 1 | 2.7(0.07-15.07) | 18 | 9.28(5.50-14.66) ^*^ |
| Diabetes mellitus | 4 | 1.27(0.35-3.24) | 1 | 0.63(0.02-3.53) | 2 | 1.5(0.18-5.43) | 7 | 1.15(0.46-2.38) |
| Alzheimer’s disease | 2 | 2.08(0.25-7.51) | 0 | 0(0.00-7.11) | 2 | 2.65(0.32-9.56) | 4 | 1.79(0.49-4.58) |
| Cardiovascular diseases | 95 | 5.18(4.19-6.33) ^*^ | 19 | 2.11(1.27-3.30) ^*^ | 8 | 1(0.43-1.97) | 122 | 3.45(2.87-4.12) ^*^ |
| Cerebrovascular diseases | 10 | 2.59(1.24-4.77) ^*^ | 3 | 1.58(0.32-4.60) | 7 | 4.01(1.61-8.27) ^*^ | 20 | 2.66(1.63-4.11) ^*^ |
| Pneumonia and influenza | 11 | 9.59(4.79-17.16) ^*^ | 2 | 3.58(0.43-12.95) | 2 | 3.74(0.45-13.51) | 15 | 6.70(3.75-11.05) ^*^ |
| COPD/ associated conditions | 20 | 8.63(5.27-13.33) ^*^ | 5 | 4.26(1.38-9.94) ^*^ | 9 | 8.33(3.81-15.82) ^*^ | 34 | 7.44(5.15-10.39) ^*^ |
| Chronic liver disease/ cirrhosis | 0 | 0(0.00-6.36) | 1 | 3.35(0.08-18.69) | 2 | 8.87(1.07-32.04) ^*^ | 3 | 2.72(0.56-7.94) |
| Nephritis, nephrotic syndrome, and nephrosis | 5 | 2.45(0.80-5.71) | 0 | 0(0.00-3.61) | 3 | 3.3(0.68-9.63) | 8 | 2.01(0.87-3.97) |
| Accidents and adverse effects of medications | 9 | 6.07(2.77-11.51) ^*^ | 2 | 2.6(0.31-9.38) | 5 | 7.71(2.50-17.99) ^*^ | 16 | 5.51(3.15-8.95) ^*^ |
| Suicide and self-inflicted injury | 0 | 0(0.00-26.07) | 1 | 14.33(0.36-79.87) | 0 | 0(0.00-72.53) | 1 | 3.81(0.10-21.25) |
| Other | 59 | 6.61(5.04-8.53) ^*^ | 24 | 5.23(3.35-7.79) ^*^ | 11 | 2.39(1.19-4.28) ^*^ | 94 | 5.19(4.20-6.35) ^*^ |

Supplementary Table 6. SMRs for each cause of death following SCLC diagnosis in black patients

* indicated p<0.05.
