# Supplementary material for: Causes of death following small cell lung cancer diagnosis: a population-based analysis
Source: BMC Pulm Med. 2022 Jul 4;22:262. doi: 10.1186/s12890-022-02053-4 (PMC9254402; doi:10.1186/s12890-022-02053-4)
Supplement: Supplementary file 7 — Additional file 7. SMRs for each cause of death following SCLC diagnosis in patients of other races. [file 12890_2022_2053_MOESM7_ESM.docx]

Supplementary Table 7. SMRs for each cause of death following SCLC diagnosis in patients of other races

|  | Deaths by time after diagnosis | | | | | |  | |
| --- | --- | --- | --- | --- | --- | --- | --- | --- |
|  | <1 y | | 1-3 y | | >3 y | | Total deaths | |
|  | Observed,  No. | SMR (95% CI) | Observed,  No. | SMR (95% CI) | Observed,  No. | SMR (95% CI) | Observed,  No. | SMR (95% CI) |
| Cause of death |  |  |  |  |  |  |  |  |
| All | 1 013 | 64.46(60.55-68.56) ^*^ | 402 | 54.03(48.87-59.58) ^*^ | 71 | 10.83(8.46-13.66) ^*^ | 1 486 | 50.01(47.50-52.62) ^*^ |
| SCLC | 898 | 781.8(731.5-834.6) ^*^ | 373 | 678.5(611.4-751.0) ^*^ | 45 | 99.52(72.59-133.17) ^*^ | 1 316 | 611.9(579.3-645.9) ^*^ |
| Other cancers | 41 | 13.21(9.48-17.92) ^*^ | 8 | 5.25(2.26-10.34) ^*^ | 3 | 2.33(0.48-6.80) | 52 | 8.79(6.56-11.52) ^*^ |
| Noncancer causes |  |  |  |  |  |  |  |  |
| Septicemia | 5 | 23.41(7.60-54.63) ^*^ | 2 | 19.56(2.37-70.67) ^*^ | 1 | 11.21(0.28-62.44) | 8 | 19.75(8.53-38.92) ^*^ |
| Infectious/ parasitic diseases  including HIV infection | 2 | 11.82(1.43-42.71) ^*^ | 0 | 0(0.00-44.54) | 1 | 14.77(0.37-82.27) | 3 | 9.38(1.94-27.42) ^*^ |
| Diabetes mellitus | 4 | 5.43(1.48-13.91) ^*^ | 0 | 0(0.00-10.25) | 1 | 3.18(0.08-17.73) | 5 | 3.55(1.15-8.27) ^*^ |
| Alzheimer’s disease | 0 | 0(0.00-12.10) | 0 | 0(0.00-25.12) | 0 | 0(0.00-22.93) | 0 | 0(0.00-6.02) |
| Cardiovascular diseases | 26 | 6.05(3.95-8.86) ^*^ | 11 | 5.60(2.79-10.01) ^*^ | 5 | 2.9(0.94-6.77) | 42 | 5.26(3.79-7.11) ^*^ |
| Cerebrovascular diseases | 1 | 0.86(0.02-4.82) | 1 | 1.87(0.05-10.41) | 3 | 6.43(1.33-18.80) ^*^ | 5 | 2.32(0.75-5.41) |
| Pneumonia and influenza | 5 | 9.49(3.08-22.16) ^*^ | 1 | 4.28(0.11-23.87) | 2 | 9.16(1.11-33.07) ^*^ | 8 | 8.18(3.53-16.11) ^*^ |
| COPD/ associated conditions | 7 | 10.06(4.05-20.73) ^*^ | 1 | 3.2(0.08-17.83) | 4 | 14.23(3.88-36.43) ^*^ | 12 | 9.31(4.81-16.26) ^*^ |
| Chronic liver disease/ cirrhosis | 1 | 5.65(0.14-31.50) | 0 | 0(0.00-39.95) | 0 | 0(0.00-49.83) | 1 | 2.91(0.07-16.23) |
| Nephritis, nephrotic syndrome, and nephrosis | 0 | 0(0.00-10.34) | 1 | 5.9(0.15-32.89) | 1 | 6.63(0.17-36.94) | 2 | 2.95(0.36-10.67) |
| Accidents and adverse effects of medications | 0 | 0(0.00-8.03) | 0 | 0(0.00-16.41) | 0 | 0(0.00-18.77) | 0 | 0(0.00-4.19) |
| Suicide and self-inflicted injury | 1 | 10.99(0.28-61.22) | 0 | 0(0.00-80.48) | 0 | 0(0.00-101.54) | 1 | 5.77(0.15-32.17) |
| Other | 22 | 9.67(6.06-14.63)^*^ | 4 | 3.66(1.00-9.36) | 5 | 4.83(1.57-11.26) ^*^ | 31 | 7.04(4.78-9.99) |

* indicated p<0.05.
