# Supplementary material for: Causes of death following small cell lung cancer diagnosis: a population-based analysis
Source: BMC Pulm Med. 2022 Jul 4;22:262. doi: 10.1186/s12890-022-02053-4 (PMC9254402; doi:10.1186/s12890-022-02053-4)
Supplement: Supplementary file 8 — Additional file 8. SMRs for each cause of death following SCLC diagnosis in patients undergoing chemotherapy. [file 12890_2022_2053_MOESM8_ESM.docx]

Supplementary Table 8. SMRs for each cause of death following SCLC diagnosis in patients undergoing chemotherapy

|  | Deaths by time after diagnosis | | | | | |  | |
| --- | --- | --- | --- | --- | --- | --- | --- | --- |
|  | <1 y | | 1-3 y | | >3 y | | Total deaths | |
|  | Observed,  No. | SMR (95% CI) | Observed,  No. | SMR (95% CI) | Observed,  No. | SMR (95% CI) | Observed,  No. | SMR (95% CI) |
| Cause of death |  |  |  |  |  |  |  |  |
| All | 17 711 | 41.94(41.33-43) ^*^ | 9 979 | 45.95(45.05-46.86) ^*^ | 1 718 | 8.68(8.27-9) ^*^ | 29 408 | 8.68(8.27-9) ^*^ |
| SCLC | 16 058 | 430.7(424.1-437.4) ^*^ | 9 163 | 490.7(480.8-500.5) ^*^ | 1 158 | 76.75(72.40-81.3) ^*^ | 26 379 | 76.75(72.40-81.3) ^*^ |
| Other cancers | 595 | 6.99(6.44-7.57) ^*^ | 319 | 7.25(6.47-8.09) ^*^ | 51 | 1.34(1.00-2) ^*^ | 965 | 1.34(1.00-2) ^*^ |
| Noncancer causes |  |  |  |  |  |  |  |  |
| Septicemia | 64 | 9.76(7.52-12.47) ^*^ | 26 | 7.51(4.91-11.01) ^*^ | 14 | 4.43(2.42-7.43) ^*^ | 104 | 4.43(2.42-7.43) ^*^ |
| Infectious/ parasitic diseases  including HIV infection | 36 | 8.21(5.75-11.37) ^*^ | 4 | 1.78(0.48-4.55) | 9 | 4.94(2.26-9.38) ^*^ | 49 | 4.94(2.26-9.38) ^*^ |
| Diabetes mellitus | 15 | 1.03(0.58-1.71) | 4 | 0.53(0.15-1.37) | 7 | 1.08(0.43-2.22) | 26 | 1.08(0.43-2.22) |
| Alzheimer’s disease | 3 | 0.31(0.06-0.92) ^*^ | 3 | 0.56(0.12-1.64) | 20 | 3.06(1.87-4.72) ^*^ | 26 | 3.06(1.87-4.72) ^*^ |
| Cardiovascular diseases | 317 | 2.84(2.54-3.18) ^*^ | 174 | 3.13(2.68-3.63) ^*^ | 116 | 2.30(1.90-2.76) ^*^ | 607 | 2.30(1.90-2.76) ^*^ |
| Cerebrovascular diseases | 46 | 2.15(1.58-2.87) ^*^ | 24 | 2.20(1.41-3.27) ^*^ | 38 | 3.68(2.60-5.05) ^*^ | 108 | 3.68(2.60-5.05) ^*^ |
| Pneumonia and influenza | 42 | 5.11(3.68-6.91) ^*^ | 14 | 3.37(1.84-5.66) ^*^ | 16 | 4.07(2.32-6.60) ^*^ | 72 | 4.07(2.32-6.60) ^*^ |
| COPD/ associated conditions | 123 | 4.29(3.57-5.12) ^*^ | 62 | 4.15(3.18-5.32) ^*^ | 129 | 9.27(7.74-11.02) ^*^ | 314 | 9.27(7.74-11.02) ^*^ |
| Chronic liver disease/ cirrhosis | 9 | 1.52(0.70-2.89) | 3 | 0.97(0.20-2.83) | 5 | 1.95(0.63-4.55) | 17 | 1.95(0.63-4.55) |
| Nephritis, nephrotic syndrome, and nephrosis | 10 | 1.21(0.58-2.22) | 12 | 2.80(1.45-4.89) ^*^ | 9 | 2.29(1.05-4.35) ^*^ | 31 | 2.29(1.05-4.35) ^*^ |
| Accidents and adverse effects of medications | 54 | 4.34(3.26-5.67) ^*^ | 27 | 4.16(2.74-6.05) ^*^ | 29 | 5.01(3.35-7.19) ^*^ | 110 | 5.01(3.35-7.19) ^*^ |
| Suicide and self-inflicted injury | 15 | 4.11(2.30-6.77) ^*^ | 7 | 3.85(1.55-7.93) ^*^ | 1 | 0.7(0.02-3.88) | 23 | 0.7(0.02-3.88) |
| Other | 324 | 4.99(4.46-5.57) ^*^ | 137 | 3.95(3.32-4.67) ^*^ | 116 | 3.35(2.77-4.02) ^*^ | 577 | 3.35(2.77-4.02) ^*^ |

* indicated p<0.05.
