# Supplementary material for: Causes of death following small cell lung cancer diagnosis: a population-based analysis
Source: BMC Pulm Med. 2022 Jul 4;22:262. doi: 10.1186/s12890-022-02053-4 (PMC9254402; doi:10.1186/s12890-022-02053-4)
Supplement: Supplementary file 9 — Additional file 9. SMRs for each cause of death following SCLC diagnosis in patients undergoing radiotherapy. [file 12890_2022_2053_MOESM9_ESM.docx]

Supplementary Table 9. SMRs for each cause of death following SCLC diagnosis in patients undergoing radiotherapy

|  | Deaths by time after diagnosis | | | | | |  | |
| --- | --- | --- | --- | --- | --- | --- | --- | --- |
|  | <1 y | | 1-3 y | | >3 y | | Total deaths | |
|  | Observed,  No. | SMR (95% CI) | Observed,  No. | SMR (95% CI) | Observed,  No. | SMR (95% CI) | Observed,  No. | SMR (95% CI) |
| Cause of death |  |  |  |  |  |  |  |  |
| All | 10 586 | 39.14(38.39-39.89) ^*^ | 6 957 | 40.20(39.26-41.16) ^*^ | 1 485 | 8.96(8.51-9.43) ^*^ | 19 028 | 31.23(30.79-31.68) ^*^ |
| SCLC | 9 625 | 400.3(392.0-408.1) ^*^ | 6 363 | 428.8(418.3-439.5) ^*^ | 991 | 76.92(72.20-81.86) ^*^ | 16 979 | 327.8(322.9-332.8) ^*^ |
| Other cancers | 364 | 6.61(5.95-7.32) ^*^ | 218 | 6.21(5.41-7.09) ^*^ | 37 | 1.15-(0.81-1.58) | 619 | 5.05(4.66-5.47) ^*^ |
| Noncancer causes |  |  |  |  |  |  |  |  |
| Septicemia | 37 | 8.76(6.17-12.07) ^*^ | 17 | 6.16(3.59-9.86) ^*^ | 13 | 4.86(2.59-8.31) ^*^ | 67 | 6.94(5.38-8.81) ^*^ |
| Infectious/ parasitic diseases  including HIV infection | 21 | 7.23(4.47-11.05) ^*^ | 4 | 2.2(0.60-5.63) | 8 | 5.14(2.22-10.13) ^*^ | 33 | 5.25(3.62-7.38) ^*^ |
| Diabetes mellitus | 9 | 0.96(0.44-1.82) | 4 | 0.67(0.18-1.71) | 6 | 1.08(0.40-2.35) | 19 | 0.91(0.55-1.42) |
| Alzheimer’s disease | 1 | 0.17(0.00-0.94) ^*^ | 3 | 0.71(0.15-2.08) | 19 | 3.67(2.21-5.73) ^*^ | 23 | 1.5(0.95-2.26) |
| Cardiovascular diseases | 155 | 2.19(1.86-2.56) ^*^ | 125 | 2.83(2.35-3.37) ^*^ | 105 | 2.51(2.05-3.03) ^*^ | 385 | 2.45(2.21-2.71) ^*^ |
| Cerebrovascular diseases | 30 | 2.22(1.50-3.17) ^*^ | 18 | 2.08(1.23-3.28) ^*^ | 37 | 4.33(3.05-5.97) ^*^ | 85 | 2.77(2.21-3.42) ^*^ |
| Pneumonia and influenza | 30 | 5.80(3.91-8.27) ^*^ | 8 | 2.42(1.04-4.77) ^*^ | 15 | 4.62(2.59-7.62) ^*^ | 53 | 4.52(3.39-5.91) ^*^ |
| COPD/ associated conditions | 77 | 4.26(3.36-5.32) ^*^ | 59 | 5.01(3.82-6.47) ^*^ | 114 | 9.81(8.09-11.78) ^*^ | 250 | 6.03(5.30-6.82) ^*^ |
| Chronic liver disease/ cirrhosis | 3 | 0.76(0.16-2.21) | 1 | 0.4(0.01-2.21) | 4 | 1.8(0.49-4.61) | 8 | 0.92(0.40-1.81) |
| Nephritis nephrotic syndrome and nephrosis | 7 | 1.33(0.53-2.73) | 9 | 2.64(1.21-5.00) ^*^ | 9 | 2.72(1.24-5.16) ^*^ | 25 | 2.08(1.35-3.07) ^*^ |
| Accidents and adverse effects of medications | 34 | 4.16(2.88-5.81) ^*^ | 21 | 3.98(2.47-6.09) ^*^ | 26 | 5.31(3.47-7.78) ^*^ | 81 | 4.41(3.51-5.49) ^*^ |
| Suicide and self-inflicted injury | 8 | 3.26(1.41-6.43) ^*^ | 6 | 4.03(1.48-8.78) ^*^ | 1 | 0.8(0.02-4.48) | 15 | 2.89(1.62-4.77) ^*^ |
| Other | 185 | 4.47(3.85-5.16) ^*^ | 101 | 3.66(2.98-4.44) ^*^ | 100 | 3.50(2.84-4.25) ^*^ | 386 | 3.95(3.57-4.37) ^*^ |

* indicated p<0.05.
