# Supplementary material for: Causes of death following small cell lung cancer diagnosis: a population-based analysis
Source: BMC Pulm Med. 2022 Jul 4;22:262. doi: 10.1186/s12890-022-02053-4 (PMC9254402; doi:10.1186/s12890-022-02053-4)
Supplement: Supplementary file 10 — Additional file 10. SMRs for each cause of death in patients with stage I-III SCLC. [file 12890_2022_2053_MOESM10_ESM.docx]

Supplementary Table 10. SMRs for each cause of death in patients with stage I-III SCLC

|  | Deaths by time after diagnosis | | | | | |  | |
| --- | --- | --- | --- | --- | --- | --- | --- | --- |
|  | <1 y | | 1-3 y | | >3 y | | Total deaths | |
|  | Observed,  No. | SMR (95% CI) | Observed,  No. | SMR (95% CI) | Observed,  No. | SMR (95% CI) | Observed,  No. | SMR (95% CI) |
| Cause of death |  |  |  |  |  |  |  |  |
| All | 7 574 | 31.37(30.67-32.09) ^*^ | 5 176 | 30.12(29.30-30.96) ^*^ | 1 430 | 8.18(7.74-8.64) ^*^ | 14 180 | 24.6(24.20-25.01) ^*^ |
| SCLC | 6 628 | 332.67(324.7-340.7) ^*^ | 4 671 | 327.89(318.5-337.4) ^*^ | 933 | 70.65(65.95-75.61) ^*^ | 12 232 | 263.59(258.9-268.3) ^*^ |
| Other cancers | 166 | 3.57(3.05-4.16) ^*^ | 135 | 3.99(3.34-4.73) ^*^ | 42 | 1.28(0.91-1.76) | 343 | 3.08(2.76-3.43) ^*^ |
| Noncancer causes |  |  |  |  |  |  |  |  |
| Septicemia | 37 | 9.88(6.96-13.62) ^*^ | 16 | 5.93(3.39-9.63) ^*^ | 16 | 5.6(3.06-9.40) ^*^ | 69 | 7.49(5.81-9.51) ^*^ |
| Infectious/ parasitic diseases  including HIV infection | 20 | 8.59(5.25-13.26) ^*^ | 3 | 1.76(0.36-5.14) | 8 | 4.87(1.96-10.03) ^*^ | 31 | 5.48(3.70-7.83) ^*^ |
| Diabetes mellitus | 15 | 1.86(1.04-3.08) ^*^ | 4 | 0.7(0.19-1.78) | 7 | 1.18(0.43-2.56) | 26 | 1.32(0.86-1.95) |
| Alzheimer’s disease | 1 | 0.15(0.00-0.85) ^*^ | 4 | 0.86(0.23-2.19) | 14 | 2.44(1.30-4.17) ^*^ | 19 | 1.08(0.64-1.71) |
| Cardiovascular diseases | 238 | 3.64(3.19-4.13) ^*^ | 123 | 2.75(2.28-3.28) ^*^ | 94 | 2.09(1.66-2.58) ^*^ | 455 | 2.96(2.69-3.25) ^*^ |
| Cerebrovascular diseases | 30 | 2.31(1.56-3.30) ^*^ | 18 | 2.03(1.20-3.21) ^*^ | 36 | 3.85(2.63-5.43) ^*^ | 84 | 2.65(2.10-3.30) ^*^ |
| Pneumonia and influenza | 33 | 6.51(4.48-9.15) ^*^ | 7 | 2.06(0.83-4.24) | 18 | 5.03(2.87-8.17) ^*^ | 58 | 4.81(3.63-6.24) ^*^ |
| COPD/ associated conditions | 142 | 8.73(7.35-10.29) ^*^ | 63 | 5.39(4.14-6.90) ^*^ | 128 | 10.51(8.68-12.62) ^*^ | 333 | 8.23(7.35-9.18) ^*^ |
| Chronic liver disease/ cirrhosis | 13 | 4.48(2.38-7.65) ^*^ | 1 | 0.45(0.01-2.49) | 3 | 1.55(0.32-4.53) | 17 | 2.4(1.40-3.84) ^*^ |
| Nephritis nephrotic syndrome and nephrosis | 14 | 2.88(1.57-4.82) ^*^ | 7 | 2.06(0.83-4.24) | 8 | 2.23(0.90-4.59) | 29 | 2.45(1.63-3.54) ^*^ |
| Accidents and adverse effects of medications | 26 | 3.88(2.53-5.68) ^*^ | 19 | 3.83(2.31-5.98) ^*^ | 30 | 6(3.95-8.73) ^*^ | 75 | 4.45(3.48-5.61) ^*^ |
| Suicide and self-inflicted injury | 14 | 7.98(4.36-13.39) ^*^ | 6 | 4.58(1.68-9.98) ^*^ | 1 | 0.93(0.02-5.16) | 21 | 5.07(3.14-7.75) ^*^ |
| Other | 197 | 5.13(4.43-5.89) ^*^ | 99 | 3.49(2.83-4.26) ^*^ | 92 | 3(2.39-3.72) ^*^ | 388 | 4.02(3.62-4.44) ^*^ |

* indicated p<0.05.
