# Supplementary material for: Causes of death following small cell lung cancer diagnosis: a population-based analysis
Source: BMC Pulm Med. 2022 Jul 4;22:262. doi: 10.1186/s12890-022-02053-4 (PMC9254402; doi:10.1186/s12890-022-02053-4)
Supplement: Supplementary file 11 — Additional file 11. SMRs for each cause of death in patients with stage IV SCLC. [file 12890_2022_2053_MOESM11_ESM.docx]

Supplementary Table 11. SMRs for each cause of death in patients with stage IV SCLC

|  | Deaths by time after diagnosis | | | | | |  | |
| --- | --- | --- | --- | --- | --- | --- | --- | --- |
|  | <1 y | | 1-3 y | | >3 y | | Total deaths | |
|  | Observed,  No. | SMR (95% CI) | Observed,  No. | SMR (95% CI) | Observed,  No. | SMR (95% CI) | Observed,  No. | SMR (95% CI) |
| Cause of death |  |  |  |  |  |  |  |  |
| All | 21 962 | 74.19(73.21-75.18) ^*^ | 5 864 | 70.93(69.12-72.77) ^*^ | 470 | 10.21(9.25-11.25) ^*^ | 28 296 | 67.38(66.60-68.18) ^*^ |
| SCLC | 19 678 | 782.11(771.2-793.1) ^*^ | 5 444 | 793.01(772.0-814.4) ^*^ | 350 | 105.03(93.6-117.4) ^*^ | 25 472 | 727.58(718.6-736.5) ^*^ |
| Other cancers | 1 143 | 19.69(18.56-20.86) ^*^ | 208 | 12.73(11.05-14.59) ^*^ | 14 | 1.61(0.83-2.81) ^*^ | 1 365 | 16.65(15.78-17.56) ^*^ |
| Noncancer causes |  |  |  |  |  |  |  |  |
| Septicemia | 69 | 15.13(11.77-19.15) ^*^ | 13 | 10.01(5.33-17.12) ^*^ | 2 | 3.14(0.38-11.36) ^*^ | 84 | 12.93(10.32-16.01) ^*^ |
| Infectious/ parasitic diseases  including HIV infection | 36 | 12.02(8.42-16.64) ^*^ | 1 | 1.19(0.03-6.62) ^*^ | 2 | 5.41(0.66-19.56) ^*^ | 39 | 9.27(6.59-12.67) ^*^ |
| Diabetes mellitus | 19 | 1.9(1.15-2.97) ^*^ | 1 | 0.36(0.01-2.00) ^*^ | 2 | 1.52(0.18-5.50) ^*^ | 22 | 1.56(0.98-2.37) |
| Alzheimer’s disease | 6 | 0.81(0.30-1.77) | 0 | 0(0.00-1.67) ^*^ | 7 | 4.24(1.55-9.22) ^*^ | 13 | 1.09(0.56-1.91) |
| Cardiovascular diseases | 355 | 4.47(4.02-4.96) ^*^ | 78 | 3.64(2.87-4.54) ^*^ | 22 | 1.81(1.09-2.82) ^*^ | 455 | 4.06(3.69-4.45) ^*^ |
| Cerebrovascular diseases | 45 | 2.94(2.15-3.94) ^*^ | 11 | 2.6(1.30-4.65) ^*^ | 7 | 2.74(1.01-5.97) ^*^ | 63 | 2.85(2.19-3.66) ^*^ |
| Pneumonia and influenza | 43 | 7.2(5.21-9.70) ^*^ | 8 | 4.94(2.13-9.73) ^*^ | 6 | 5.92(1.92-13.81) ^*^ | 57 | 6.64(5.02-8.62) ^*^ |
| COPD/ associated conditions | 150 | 7.56(6.40-8.88) ^*^ | 21 | 3.77(2.34-5.77) ^*^ | 21 | 6.56(3.89-10.36) ^*^ | 192 | 6.72(5.79-7.75) ^*^ |
| Chronic liver disease/ cirrhosis | 2 | 0.51(0.06-1.83) | 3 | 2.66(0.55-7.76) ^*^ | 1 | 2.1(0.05-11.68) ^*^ | 6 | 1.08(0.40-2.35) |
| Nephritis nephrotic syndrome and nephrosis | 17 | 2.91(1.69-4.66) ^*^ | 5 | 3.06(0.99-7.14) ^*^ | 3 | 3.67(0.76-10.74) ^*^ | 25 | 3.01(1.95-4.45) ^*^ |
| Accidents and adverse effects of medications | 44 | 5.11(3.71-6.86) ^*^ | 9 | 3.68(1.68-6.99) ^*^ | 5 | 3.44(0.94-8.82) ^*^ | 58 | 4.66(3.53-6.04) ^*^ |
| Suicide and self-inflicted injury | 9 | 3.65(1.67-6.92) ^*^ | 2 | 2.99(0.36-10.79) ^*^ | 1 | 3.7(0.09-20.59) ^*^ | 13 | 3.52(1.82-6.15) ^*^ |
| Other | 346 | 7.45(6.68-8.28) ^*^ | 59 | 4.4(3.35-5.68) ^*^ | 27 | 3.33(2.13-4.95) ^*^ | 432 | 6.4(5.81-7.03) ^*^ |

* indicated p<0.05.
